# Continuous Glucose Monitoring With Low-Carbohydrate Diet Coaching in Adults With Prediabetes: Mixed Methods Pilot Study

**DOI:** 10.2196/21551

**Published:** 2020-12-16

**Authors:** Olivia Yost, Melissa DeJonckheere, Spring Stonebraker, Grace Ling, Lorraine Buis, Rodica Pop-Busui, Noa Kim, Kara Mizokami-Stout, Caroline Richardson

**Affiliations:** 1 Department of Family Medicine University of Michigan Ann Arbor, MI United States; 2 Institute for Healthcare Policy and Innovation University of Michigan Ann Arbor, MI United States; 3 Division of Metabolism, Endocrinology and Diabetes Department of Internal Medicine University of Michigan Ann Arbor, MI United States

**Keywords:** prediabetes, type 2 diabetes mellitus, prevention & control, low carbohydrate diet, diet modification, blood glucose self-monitoring

## Abstract

**Background:**

Type 2 diabetes mellitus (T2DM) is preventable; however, few patients with prediabetes participate in prevention programs. The use of user-friendly continuous glucose monitors (CGMs) with low-carbohydrate diet coaching is a novel strategy to prevent T2DM.

**Objective:**

This study aims to determine the patient satisfaction and feasibility of an intervention combining CGM use and low-carbohydrate diet coaching in patients with prediabetes to drive dietary behavior change.

**Methods:**

We conducted a mixed methods, single-arm pilot and feasibility study at a suburban family medicine clinic. A total of 15 adults with prediabetes with hemoglobin A_1c_ (HbA_1c_) levels between 5.7% and 6.4% and a BMI >30 kg/m^2^ were recruited to participate. The intervention and assessments took place during 3 in-person study visits and 2 qualitative phone interviews (3 weeks and 6 months after the intervention). During visit 1, participants were asked to wear a CGM and complete a food intake and craving log for 10 days. During visit 2, the food intake and craving log along with the CGM results of the participants were reviewed and the participants received low-carbohydrate diet coaching, including learning about carbohydrates and personalized feedback. A second CGM sensor, with the ability to scan and record glucose trends, was placed, and the participants logged their food intake and cravings as they attempted to reduce their total carbohydrate intake (<100 g/day). During visit 3, the participants reviewed their CGM and log data. The primary outcome was satisfaction with the use of CGM and low-carbohydrate diet. The secondary outcomes included feasibility, weight, and HbA_1c_ change, and percentage of time spent in hyperglycemia. Changes in attitudes and risk perception of developing diabetes were also assessed.

**Results:**

The overall satisfaction rate of our intervention was 93%. The intervention induced a weight reduction of 1.4 lb (*P*=.02) and a reduction of HbA_1c_ levels by 0.71% (*P*<.001) since enrollment. Although not significantly, the percentage of time above glucose goal and average daily glucose levels decreased slightly during the study period. Qualitative interview themes indicated no major barriers to CGM use; the acceptance of a low-carbohydrate diet; and that CGMs helped to visualize the impact of carbohydrates on the body, driving dietary changes.

**Conclusions:**

The use of CGMs and low-carbohydrate diet coaching to drive dietary changes in patients with prediabetes is feasible and acceptable to patients. This novel method merits further exploration, as the preliminary data indicate that combining CGM use with low-carbohydrate diet coaching drives dietary changes, which may ultimately prevent T2DM.

## Introduction

### Background

Type 2 diabetes mellitus (T2DM) is a preventable disease; however, most of the 84 million adults in the United States who have prediabetes do not participate in evidence-based prevention programs [1-3]. Although the Diabetes Prevention Program (DPP) study found that people with prediabetes can reduce their risk of developing T2DM by 58% through participation in an intensive lifestyle modification program [1], personal and logistical barriers limit participation. Innovative, low-cost methods to prevent T2DM in the primary care setting are needed.

The New American Diabetes Association care guidelines [4] state that low-carbohydrate diet plans may result in improved glycemia [5] and help patients with prediabetes in decreasing postprandial glucose spikes that are frequently followed by crashes and cravings. Low-carbohydrate diets have shown positive effects for the prevention of prediabetes [6,7] and management of T2DM [8,9]. However, patients with prediabetes may lack sufficient motivation and support [7] or knowledge to adopt and maintain a low-carbohydrate diet. Although limited, research has supported the use of health coaching interventions for adults with prediabetes and type 2 diabetes to increase knowledge, increase motivation, and support long-term behavioral changes. For example, health coaching has been used to improve diet quality, exercise adherence, diabetes self-efficacy, diabetes empowerment, social support, and reduce diabetes distress in individuals with type 2 diabetes [10-13]. For adults with prediabetes, DeJesus et al [14] found that a 12-week wellness coaching program improved physical activity, healthy eating behaviors, self-efficacy, and quality of life. Further research is needed to specifically examine the use of health coaching that emphasizes a low-carbohydrate diet for individuals with prediabetes.

Simultaneously, the use of new technology may be another useful strategy for improving engagement and adherence to T2DM prevention programs. In a meta-analysis by Bian et al [15], technology-mediated interventions were shown to lead to clinically significant weight loss in individuals at risk for T2DM, particularly when combined with a DPP model. New low-cost and user-friendly continuous glucose monitors (CGMs) have made it feasible to use CGM technology for diabetes prevention. Although CGMs are primarily used in patients with type 1 diabetes to adjust the insulin dosage and prevent hypoglycemia, more recently, CGMs have also been prescribed for patients with T2DM who face challenges in diabetes management [16]. However, there is a lack of research on CGMs as a prevention or behavior modification tool [17]. In a recent review, Ehrhardt et al [17] described 2 pilot studies examining the impact of CGMs as a behavior modification tool to improve physical activity [18,19]. However, the impact of CGMs on dietary behavior remains to be unknown [18,19]. As CGMs offer their wearers personalized feedback about the effect of dietary choices on blood glucose trends, the use of CGMs could be a viable strategy for dietary interventions that seek to reduce glycemic variability, which is known to increase the risk of adverse outcomes [20].

### Objectives

To address these 2 important and related gaps in the literature, we developed a novel approach of combining real-time feedback from a CGM with low-carbohydrate dietary coaching. As low-carbohydrate diets are likely to reduce postprandial glucose spikes [21], participants will be able to see the corresponding flattening of blood glucose peaks and crashes as they modify their diet. This integrated approach has the potential to make individuals with prediabetes aware about the impact of carbohydrates on their blood glucose levels, thereby supporting behavior change with personalized feedback. Thus, this pilot study aims to determine the feasibility of combining low-carbohydrate diet coaching with real-time CGM feedback in patients with prediabetes to drive behavior change and reinforce low-carbohydrate diet adherence.

## Methods

**Figure 1:**
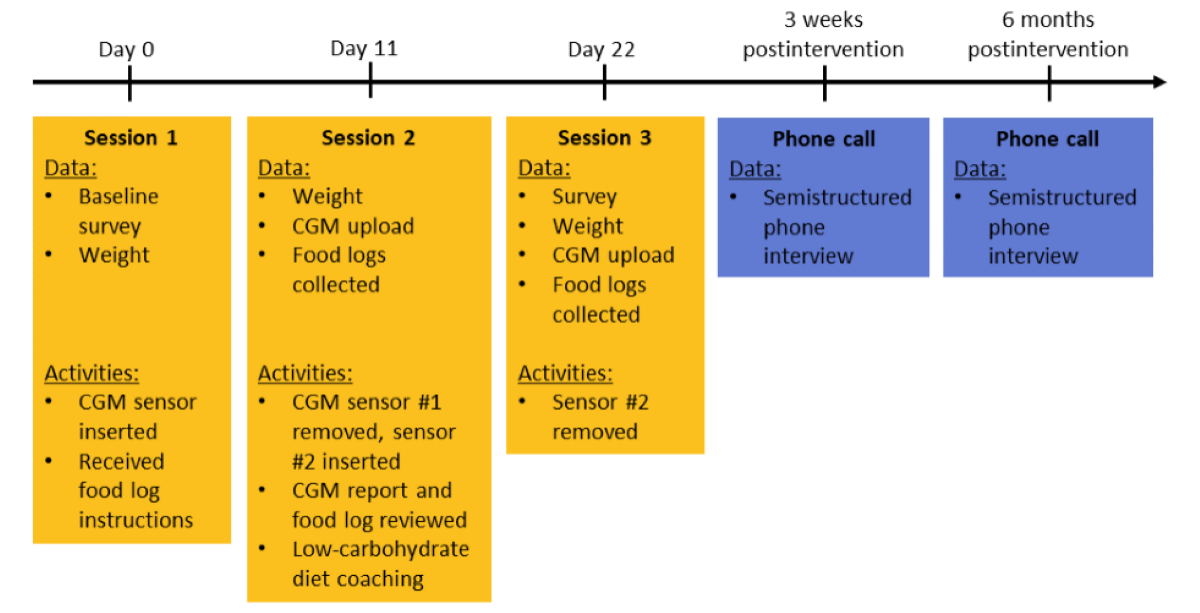
Pilot and feasibility design diagram. CGM: continuous glucose monitor.

This was a mixed methods, single-arm, pilot and feasibility study with 15 participants. The participants attended 3 sessions with a study coordinator, which included coaching on a low-carbohydrate diet. The study coordinator for this study was a certified medical assistant. She was provided with instructions on how to implement the intervention and provide low-carbohydrate diet coaching. CGMs were provided at 2 study visits. The primary outcome was participant satisfaction with the intervention: low-carbohydrate diet coaching with continuous glucose monitoring. Secondary outcomes included feasibility, weight change, the percentage of time spent in hyperglycemia, side effects of CGM wear, and use of CGMs. Figure 1 shows the overall design diagram.

### Subjects and Recruitment

Eligible participants were identified from a southeast Michigan Family Medicine office by searching existing electronic health record data. Participants were required to be of 21 years of age or above, have a BMI >30 kg/m^2^, and have an HbA_1c_ level between 5.7% and 6.4% in the last year. Participants were excluded if they were on diabetes medications (eg, metformin), previously had bariatric surgery, were pregnant or breastfeeding, or classified themselves as vegan or vegetarian. In addition, participants were required to be interested in changing their diet to improve their health, have a phone, and speak, read, and write in English.

Eligible participants received a letter explaining the study and its requirements with an opt-out postcard. Those who did not opt out were contacted via phone with further information. Interested and eligible participants met the study coordinator at the family medicine office to be enrolled for their baseline visit. All subjects signed a written consent, and the study was approved by the University of Michigan Institutional Review Board.

### Intervention

Participants attended 3 sessions with the study coordinator (Figure 1). At visit 1, participants received information on CGM use and an Abbott Libre Pro sensor was applied to their arm. At the time of the study, the Abbott Libre Pro sensor was able to record data for a total of 10 days before the sensor period ended and the sensor needed to be replaced. The sensor did not record any blood glucose values during the wearing period. Participants were asked to wear the sensor for the 10-day sensor period and to complete a food log, documenting what they consumed, their fatigue levels, and their cravings 2 hours after eating. Participants received a copy of the book *Always Hungry* [22] that describes a low-carbohydrate diet program.

At visit 2 (11 days later), participants returned for a one-on-one low-carbohydrate diet coaching session with the study coordinator. The first sensor was removed, and data were uploaded, reviewed, and printed for the participant. Participants received coaching on low-carbohydrate diets, which included a comparison of their completed food logs with the CGM data, information on the recommended carbohydrate intake, and resources to determine the carbohydrate content of popular foods. They were asked to have a low-carbohydrate diet (less than 100 g per day) for the duration that they wore the second sensor, which also lasted for 10 days. Participants were advised to increase their protein and water intake. Participants also received additional training on CGM use, and the Abbott Libre personal sensor (which allows viewing real-time glucose data) was applied to their arm. 

At visit 3 (11 days later), participants had the second sensor removed and data were uploaded and printed for review. Participants reviewed their food logs with their CGM trends with the study coordinator. Participants were given compensation of US $25.

### Quantitative Methods

#### Data Collection

##### Outcome Measures

###### Satisfaction, Feasibility, and Acceptability

Participant satisfaction was measured through postintervention surveys as well as through qualitative interviews. Participants were asked, on a 5-point Likert scale, to indicate (1) how satisfied they were with the intervention (low-carbohydrate diet with CGM use), (2) how likely they were to recommend a low-carbohydrate diet to others with prediabetes, (3) how likely they were to recommend a CGM to a family member or friend with prediabetes, and (4) how likely they were to purchase a CGM to test their blood glucose. The last item did not include specific information about the cost of a CGM or availability of insurance coverage. Feasibility and acceptability were measured based on successful recruitment and enrollment of 15 study participants, CGM wear times of 20 to 22 days in total, CGM data retrieval, and completion of food logs. Interviews explored participants’ experiences with the low-carbohydrate diet, coaching, CGM use, and any barriers the participants faced.

###### Weight

At each visit, participant weight was measured in pounds using a standing scale, without shoes and heavy clothing.

###### Estimated HbA_1c_, Average Daily Glucose, and Percentage of Time Spent in Hyperglycemia

All glucose-related variables were calculated using Abbott Freestyle Libre CGM software. The estimated HbA_1c_ level was calculated using the Nathan formula [23]. The average daily glucose was calculated as the mean of all the glucose sensor readings for a 24-hour period. The percentage of time spent in hyperglycemia was defined and calculated as the period in which glucose levels were >140 mg/dL for over 24 hours.

###### Perceived Risk of Diabetes

We measured the perceived risk of diabetes by asking questions developed from the KORA FF4 study [24] pre- and postintervention. Items included estimates of the risk of participants having diabetes at present (6-point Likert scale from negligible to very high), developing diabetes in the next 3 years (yes, no, and I do not know), and whether diabetes is a serious disease (4-point Likert scale from not serious to very serious).

###### Risk Perception Survey for Developing Diabetes

Risk perception for developing diabetes was measured using the risk perception survey for developing diabetes (RPS-DD) preintervention and postintervention [25]. A total of 3 subscales were included: personal control subscale (4 items), optimistic bias subscale (2 items), and worry subscale (2 items). Each item was presented as a statement and scored on a 4-point Likert scale (1=strongly agree; 4=strongly disagree). Subscale scores and a composite score were calculated for each participant, with higher scores indicating a higher level of the assessed underlying construct: more personal control, optimistic bias, and worry.

###### Modified Weight Loss Readiness Test II

Participants were asked questions based on a modified form of the Weight Loss Readiness Test II motivation questions, which were previously used in a pragmatic clinical trial of the DPP for Veterans Health Administration patients with prediabetes [26]. Participants rated how motivated they were to lose weight, exercise, eat a healthy diet, and avoid developing diabetes. Items were scored on a Likert scale ranging from 1 (*very motivated*) to 5 (*not motivated at all*).

###### Data Analysis

We performed descriptive statistical analyses for demographic variables. For categorical variables (eg, satisfaction, feasibility, and acceptability of the intervention), we calculated frequencies for each category. For all continuous variables, we conducted paired *t* tests to examine changes from baseline to postintervention. All statistical analyses were conducted using STATA statistical software (StataCorp) [27].

#### Qualitative Methods

##### Data Collection

We conducted semistructured interviews [28] with participants at 2 points: approximately 3 weeks after the intervention and 6 months after the intervention. All participants were invited to complete both interviews. The interview guide was designed to elicit participant experiences across several domains, including living with prediabetes, efforts to reduce risk of developing diabetes, experience with the low-carbohydrate diet and coaching, use of CGMs, and intentions moving forward. Interviews were conducted by a qualitative methodologist (MD) and a family medicine resident (OY) trained and mentored in qualitative research. All interviews were conducted via phone or web conference and were audio-recorded.

##### Data Analysis

Audio recordings were professionally transcribed. We conducted 2 inductive, thematic analyses [29] to understand participant perspectives during the intervention. First, we analyzed transcripts from 3 weeks after the intervention. Two investigators (OY and MD) reviewed the first 2 transcripts to develop codes that represented meaningful concepts in the data. Codes were agreed upon and then applied to 2 additional transcripts. We discussed the coding scheme to ensure that codes were consistently applied across transcripts and discrepancies were resolved. The remaining transcripts were coded by both investigators. Next, we summarized the content of each code by reviewing all data segments assigned to an individual code. The code summarizes detailed variation within each code and illustrative quotes. After creating the summaries, we developed themes that incorporated multiple, interrelated codes that were reported by more than one participant. The same process was completed for the interviews conducted 6 months after the intervention.

##### Mixed Methods Analysis

The purpose of the mixed methods analysis was to develop hypotheses that may explain the differences in the intervention outcomes and to identify focus areas for future iterations of the intervention. To integrate the quantitative and qualitative approaches, we compared the thematic results of different groups of participants based on significant quantitative results: reduction in HbA_1c_ levels and weight loss. First, we compared the experiences (in the form of qualitative themes and quotes) reported by participants who had a less-than-average reduction in HbA_1c_ levels with those reported by the participants who had an above-average reduction in HbA_1c_ levels. Second, we compared the experiences of those with less-than-average weight loss with those with greater-than-average weight loss. For both comparisons, we created joint displays, a visual strategy that can be used to bring together quantitative and qualitative results for a mixed methods analysis and interpretation [30,31].

## Results

A total of 15 participants were enrolled in this study. The mean age was 54.5 (SD 9.1) years. Participants had a mean enrollment HbA_1c_ level of 5.9% (SD 0.23), BMI of 35.8 (SD 4.7) kg/m^2^, and starting weight of 232.7 (SD 45.1) lbs. Of the total 15 participants, 10 (67%) were women, 11 (73%) identified as White, and 4 (27%) identified as African American. Table 1 shows the participant demographics.

**Table 1:** Participant demographics of the pilot feasibility study (N=15).

Characteristics		Values
Age (years), mean (SD)		54.5 (9.1)
Enrollment HbA_1c_^a^ (%), mean (SD)		5.9 (0.23)
BMI (kg/m^2^), mean (SD)		35.8 (4.7)
Starting weight (lbs), mean (SD)		232.7 (45.1)
**Gender, n (%)**		
	Male	5 (33)
	Female	10 (67)
**Race, n (%)**		
	White	11 (73)
	African American	4 (27)
**Education, n (%)**		
	Completed high school	15 (100)
	Bachelor’s degree	8 (53)

^a^HbA_1c_: hemoglobin A_1c_.

### Feasibility and Satisfaction Results

All 15 participants wore both sensor 1 and sensor 2 for an average of 9.8 (SD 1.9) and 9.6 (SD 0.8) days, respectively. Of the total, 80% (12/15) of the participants completed food log number 1 and 87% (13/15) completed food log number 2. All participants attempted a low-carbohydrate diet during the intervention. Of the total, 13 participants completed both interviews. Of the total, 93% (14/15) of the participants reported satisfaction with the intervention, whereas 7% (1/15) reported neutral satisfaction.

When asked if they would recommend a low-carbohydrate diet to others with prediabetes, 100% (15/15) were extremely likely (n=12) or likely to (n=3) recommend. A total of 10 participants said they were extremely likely to recommend wearing a CGM to a family member or a friend with prediabetes, whereas 4 participants said they were likely, and 1 reported neither likely nor unlikely. When asked how likely they were to buy a CGM to test their blood glucose levels, 3 reported extremely likely, 6 likely, 3 neutral, 2 unlikely, and 1 did not answer. There were no major adverse events reported for the duration of this study with CGM use.

### Quantitative Results

Results were significant for the reduction in HbA_1c_ levels from the final estimated HbA_1c_ level to HbA_1c_ level measured at the time of enrollment (–0.71%; *P*<.001) and weight change from the second to final visit (–1.4 lb; *P*=.02). The percentage of time spent in hyperglycemia (>140 mg/dl) and average daily glucose were not significant but tended to decrease during the intervention period (Table 2). Pre- and posttest scores for the 3 measures presented in Table 3.

**Table 2:** Changes in hemoglobin A_1c_, weight, and blood glucose.

Measure		Mean (SD)		SE		*P* value	
**Hemoglobin A_1c_(%)**							
	Enrollment^a^		5.9 (0.23)		0.06		N/A^b^
	Sensor 1^c^		5.2 (0.38)		0.10		N/A
	Sensor 2^c^		5.2 (0.37)		0.10		N/A
	Δ^d^: Sensor 2—Enrollment		–0.71 (0.46)		0.12		<.001
	Δ: Sensor 2—Sensor 1		–0.01 (0.31)		0.08		.87
**Weight (lbs)**							
	Visit 1 (starting weight)		232.7 (45.1)		11.7		N/A
	Visit 2		233.2 (46.2)		11.9		N/A
	Visit 3		231.8 (45.9)		11.8		N/A
	Δ: Visit 3—Visit 1		–0.89 (2.94)		0.76		.26
	Δ: Visit 3—Visit 2		–1.41 (2.18)		0.56		.02
**Average daily glucose (mg/dL)**							
	Sensor 1		103.8 (10.6)		2.8		N/A
	Sensor 2		102.9 (10.9)		2.9		N/A
	Δ: Sensor 2—Sensor 1		–0.93 (8)		2.1		.67
**Time spent in hyperglycemia^e^(%)**							
	Sensor 1		7.1 (7.9)		2.1		N/A
	Sensor 2		4.5 (5.6)		1.5		N/A
	Δ: Sensor 2—Sensor 1		–2.6 (6.5)		1.7		.16

^a^Measured hemoglobin A_1c_ (HbA_1c)_ by clinical laboratory.

^b^N/A: not applicable.

^c^HbA_1c_ estimated from continuous glucose monitor data.

^d^Delta or difference.

^e^Glucose >140 mg/dL.

**Table 3:** Pre- and postscores of the KORA FF4, RPS-DD, and modified Weight Loss Readiness Test II.

Tool		Participants, n (%)	Mean prescores, mean (SD)	Mean postscores, mean (SD)	Mean delta	SE	*P* value
**KORA FF4**							
	Diabetes risk at present^a^	14 (93)	4.21 (1.31)	2.71 (1.20)	–1.5	0.402	.002
	Risk of developing diabetes in the next 3 years^b^	8 (53)	1.13 (0.35)	1.63 (0.52)	0.5	0.19	.003
	How serious of a disease is diabetes?^c^	14 (93)	3.64 (0.74)	3.78 (0.43)	–0.14	0.21	.50
**RPS-DD^d^**							
	Personal control	13 (87)	14.31 (1.60)	14.54 (2.07)	0.23	0.30	.46
	Optimistic bias	15 (100)	3.33 (1.05)	4 (1)	0.67	0.33	.06
	Worry	10 (67)	4.7 (1.42)	5.3 (1.64)	0.6	0.40	.17
	Composite	8 (53)	7.63 (0.90)	7.92 (1.02)	0.29	0.21	.21
**Modified Weight Loss Readiness Test II^e^**							
	Lose weight	15 (100)	1.53 (0.52)	1.4 (0.63)	–0.13	0.19	.49
	Exercise	15 (100)	2.13 (1.06)	2 (1)	–0.13	0.19	.49
	Have a healthy diet	15 (100)	1.4 (0.51)	1.33 (0.62)	–0.067	0.15	.67
	Avoid developing diabetes	15 (100)	1.4 (0.63)	1.33 (0.72)	–0.067	0.12	.58

^a^Likert scale of 1-6 with a higher score indicating higher risk.

^b^Scored 1=yes, 2=no. Answers of I don’t know were excluded from analysis.

^c^Likert scale of 1-4 with a higher score indicating more seriousness.

^d^RPS-DD: risk perception survey for developing diabetes. Likert scale from 1-4 with a higher score indicating a higher level of the assessed underlying construct.

^e^Likert scale of 1-5 with a lower score indicating increased motivation.

### Perceived Risk of Diabetes

The estimated risk of developing disease at the present moment decreased during the intervention (mean 4.21, SD 1.31 vs 2.71, SD 1.20; n=14; *P*=.002). Participants believed that their risk of developing diabetes in the next 3 years was less following the intervention (1.13, SD 0.35 vs 1.63, SD 0.52; n=8; *P*=.003). The perception of the seriousness of diabetes among participants was not significantly different following the intervention (n=14; *P*=.50).

### Risk Perception of Developing Diabetes

Composite scores for the risk of developing diabetes increased from 7.63 to 7.92 during the intervention. Participants’ sense of personal control over their health and diabetes was not significantly different before and after the intervention (n=13; mean 14.31, SD 1.60) and 14.54, SD 2.07), respectively; n=13; *P*=.46). Optimistic bias average scores increased from 3.33 (SD 1.05) to 4.00 (SD 1); n=15; *P*=.06 and approached significance. This increase corresponds to participants who believed that they are less likely to develop T2DM than their peers following the intervention. The change in worry about developing diabetes was not significant (mean 4.7, SD 1.42 vs 5.3, SD 1.64; n=8; *P*=.17).

### Readiness to Lose Weight, Exercise, Eat Healthy, and Avoid Diabetes

Participant motivation did not change significantly; however, it trended toward increased motivation postintervention to lose weight, exercise, eat a healthy diet, and avoid getting diabetes.

### Qualitative Results

A total of 13 participants completed 2 semistructured interviews at approximately 3 weeks and 6 months after the intervention, whereas 2 participants declined to attend the interview. The thematic analysis resulted in 3 themes that spanned both time points: (1) participants reported no major barriers to CGM use, (2) all participants attempted a low-carbohydrate diet, and (3) CGMs helped participants to visualize the impact of carbohydrates on glucose trends, inciting dietary changes (Textbox 1 lists the themes and related participant experiences).

**Themes and related participant experiences during the pilot and feasibility study.**

Participants reported no major barriers to continuous glucose monitor (CGM) use

- Reported CGMs were comfortable to wear
- Described no adverse effects
- Reported barriers to CGM use outside of the intervention

All participants attempted a low-carbohydrate diet

- Experienced positive physical effects
- Described barriers to diet
- Modified the diet for sustainability

CGMs helped to visualize the impact of carbohydrates on glucose trends, inciting dietary changes

- Preferred seeing glucose trends in real time
- Reflected on impact of carbohydrates on glucose levels
- Felt reassured by trends in CGM data
- Felt unsure of the need for CGM outside of the intervention

#### Theme 1: Participants Reported No Major Barriers to CGM Use

Across participants, wearing the CGM did not cause adverse effects. Participants described that CGM insertion was relatively painless and that they did not feel discomfort wearing it (eg, “I mean after I got used to it, it was fine. It didn’t bother me at all,” [Participant 105, Interview 1]). However, a few participants noted that the CGM sensor could get in the way at times (eg, getting caught on clothes or pushed against throughout the day), but this was not a major barrier that prevented its use. For example, one participant reflected the following:

I didn’t even realize it was there. I think, probably when I would get out of the shower, or different clothes that I’d be putting on or taking off made me aware of it being there. But, other than that, I completely forgot about it.

Participant 179, Interview 1

In a social setting, a few participants shared that peers noticed them wearing the sensor. One participant explained the following:

[Others would notice it] when I go to water therapy. So it was like what’s that? You know. Then I would tell ‘em. So yeah, other than that, you know, just people curious as far as what was the function of it.

Participant 222, Interview 1

As a result, 1 participant reported that they would not be as comfortable wearing the sensor during the summer when their arms would be exposed.

Participants reported that CGM use was less invasive and painful compared with using a glucometer to monitor their glucose levels. For example:

At first I could feel that [the sensor] was there. Then, after a little bit, I didn’t even notice it. I liked that they had [the CGM] and I wouldn’t need to prick my finger all the damn time.

Participant 144, Interview 1

In the interviews conducted 6 months after the intervention, participants continued to report that wearing a CGM is an easy and comfortable way to monitor their blood glucose levels. Although they did not continue wearing a CGM after the intervention, 1 participant expressed their preference for wearing the sensor:

[The monitor] was a very easy way for people to check their sugar counts. It was very good to monitor it that way. I think some people think it will really feel terrible on their arm, or they wouldn’t want that. I heard a couple comments people said to me, and I said, “I don’t even feel it” (laugh). I mean it’s just an easy way to do it. I would wear one if I truly had to.

Participant 102, Interview 2

One participant, who received a prescription and wore the sensor after the intervention, further described how the CGM was a discrete option for blood glucose monitoring and how it was suitable for her lifestyle:

Once I got it [the CGM] and read through the directions, I’m like oh, there’s an app; I could have just used that, and not have to worry about it. And that’s what I used when I traveled was just the app. It’s nice too because if you’re in a business meeting and nobody knows you have it on, you just kind of put your phone up next to it, and you know what it

your glucose] is. Nobody knows; it’s discrete. [Participant 193, Interview 2

However, the participant above was the only participant who reported obtaining another CGM after the intervention. Others reported that they did not get a CGM after the intervention for various reasons. One participant tried to get a CGM, but their insurance did not cover it, whereas others stated that they did not know it was an option for them:

I felt like the insurance wouldn’t cover it because I wasn’t really in that range

prediabetes] anymore. [Participant 199, Interview 2

Several described not needing to use a CGM after the intervention because their participation helped them to understand how to eat to prevent hyperglycemia or prevent diabetes:

I considered [getting a prescription]. I noticed when I was doing the monitoring, my numbers were pretty good. I remember the eating that I did when I was on the monitor, so I stuck to that diet in hopes that my numbers would be about the same.

Participant 169, Interview 2

However, some participants were willing to reconsider wearing a CGM sensor if they needed to *get back on track* to prevent diabetes or if they were diagnosed with diabetes:

Right now I think as long as I’m doing 6 month A_1c_ checkups, and it’s going lower, that I’m okay that I don’t feel I need to continuously monitor it. But if it starts to trend like it was coming back up, I think I would wear one, just to try to get back on track and get it lower. And keep me off medicine.

Participant 193, Interview 2

#### Theme 2: All Participants Attempted a Low-Carbohydrate Diet

All participants reported reducing their carbohydrate intake during the intervention. Overall, participants reported consuming more protein and vegetables and reducing simple carbohydrates. One participant described the dietary changes they made during the intervention:

For dinner was usually chicken, and a salad, and more vegetables. And every now and then, my sister would make some pasta, but it wasn’t always the big blue bowl that I would eat, it would be the little small bowl. I really worked on not eating what I was used to eating.

Participant 231, Interview 1

Some reported positive effects such as feeling better, sleeping better, and having more energy:

Physically [I] definitely felt better. I don’t think I was as tired. I definitely felt I didn’t have a lot of the swings that I normally have in terms of being tired or lethargic.

Participant 199, Interview 1

Those who were able to maintain the low-carbohydrate diet continued to see positive changes that motivated them to sustain the dietary changes, including increased weight loss, increased energy, improved sleep, and lowered HbA_1c_:

When I was a little heavier, I did not like the way I looked or felt. So I’ve actually enjoyed and appreciated being a lot slimmer. Between feeling better and looking better, that keeps me motivated to keep up the same type of eating habits. Not to mention, in the time my A_1c_ has continued to drop. So, my family has a history of diabetes, and I do not want to be included in that list if I can help it.

Participant 169, Interview 2

#### Barriers to a Low-Carbohydrate Diet

Despite trying the diet, there were some barriers to low-carbohydrate diets reported across participants. Some participants described feeling bored with food choices, whereas others still had to give up their old eating habits. For example, 1 participant reflected on the diet:

Probably after about 4 days it was a lot harder to do, just because you kinda get sick of all of the same choices I s’pose. I don’t know, it’s just really protein heavy, and it just got a little bit… my mind knew better, but my mouth wanted certain things.

Participant 199, Interview 1

Being creative with meals is difficult. I was pretty much protein vegetable, protein vegetable, protein vegetable, you know. It gets boring after a while, so I think variety’s important.

Participant 219, Interview 1

Participants also emphasized that to maintain a low-carbohydrate diet, it is imperative to plan ahead and have healthy choices available:

I’m tryin’ you know, like I said every day to figure out what my meals are gonna be. Instead of just eating anything in the refrigerator. And before, whatever is in the refrigerator sometimes wasn’t the healthiest. I mean it’s not easy by no means. Eating healthy is not easy.

Participant 231, Interview 1

Some participants also reported that they felt the need to get rid of old eating habits and continue to make good choices, particularly when having cravings:

Oh, nothin’ really made it difficult. Just tryin’ to, maintain that, every day you know…We got a lot a food in the house, so every day is just makin’ the right choice.

Participant 222, Interview 1

Others described the difficulty of making healthy choices when people around them, including family members, friends, and colleagues, do not maintain the same lifestyle. This was particularly salient around winter holidays.

#### Maintenance and Modification of a Low-Carbohydrate Diet

In the interviews conducted 6 months after the intervention, the majority of the participants reported that they were trying to maintain their diet after the intervention. Most participants had modified the diet to be “less strict” but “still healthy.” For example, participants reported avoiding processed foods, eating more fruits and vegetables, and being more carb conscious. Many participants described intermittently straying from the low-carbohydrate diet before returning to a less restrictive version:

Well just last week I started on it again. Not as strict as I was before. But, I said no, I have to start doing something again. So, I just kind of started it. I tried to cut out all carbs, but now I’m allowing myself a few more carbs. So, you know, maybe it won’t be so restrictive for me.

Participant 102, Interview 2

Participants reported similar barriers in the interviews conducted 6 months after the intervention, including having to break old habits and dealing with cravings for higher-carbohydrate and processed foods:

Well, put it this way, I’m not very successful at following the low carb, high protein diet. And so yeah, I fell off it. I had done that once before. It seems difficult, for whatever reason. You know you say to yourself I wanna change somethin’, so you eat, and then, you know I want a hamburger (Laugh) So, you just kinda fall off. And it’s just the story of my life, lack of discipline.

Participant 219, Interview 2

In addition, some participants reported that maintaining a low-carbohydrate diet was too expensive. One participant explained the following:

Believe it or not, it’s actually cheaper to eat carbs than it is to eat healthy . . . Fruits and vegetables are so much more expensive now, and, it’s just easier to grab a bag a chips or something.

Participant 179, Interview 2

Yet, most of the participants described trying to continue eating better, even if every choice was not low in carbohydrates.

#### Theme 3: CGMs Helped to Visualize the Impact of Carbohydrates on Glucose Trends, Driving Dietary Changes

Overall, participants were able to use the CGM data to help them understand fluctuations in blood glucose trends. During the first week of the study, participants reviewed CGM data alongside their food logs with the study coordinator. Participants were able to visualize the impact of food on their blood glucose levels and understand trends.

In the first interview, 1 participant reported that they learned how their regular eating habits affected their glucose levels:

I was fascinated, and very excited to see the results of it. The first week I just continued to eat normally, and, I was able to see... what I was doing, and it was horrible. I mean I had mountains and valleys. It was just up and down all day long, based on the way that I ate normally every single day.

Participant 213, Interview 1

**Figure 2:**
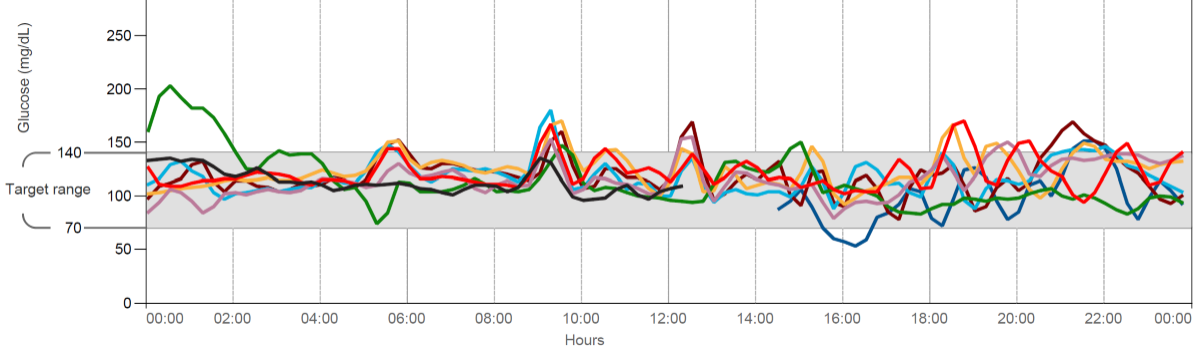
Example of continuous glucose monitor data downloaded from Abbott software and reviewed with participants during low-carbohydrate diet coaching with the study coordinator. Each individual colored line represents the collected glucose data for a distinct 24-hour period.

The experience of this participant in visualizing peaks and valleys is evident in the daily patterns available from their CGM for the first week (Figure 2).

In the second week, participants were able to use the CGM scanner to see their real-time blood glucose levels. Participants unanimously preferred seeing the data in real time to compare the changes in their blood glucose with the foods they had eaten. One participant explained the following:

I was more conscious of it, I was able to see in real time what I was eating was doing to me. You can listen to dieticians and all this other stuff, and if you can’t really see it, you don’t know, you don’t realize.

Participant 141, Interview 1

**Figure 3:**
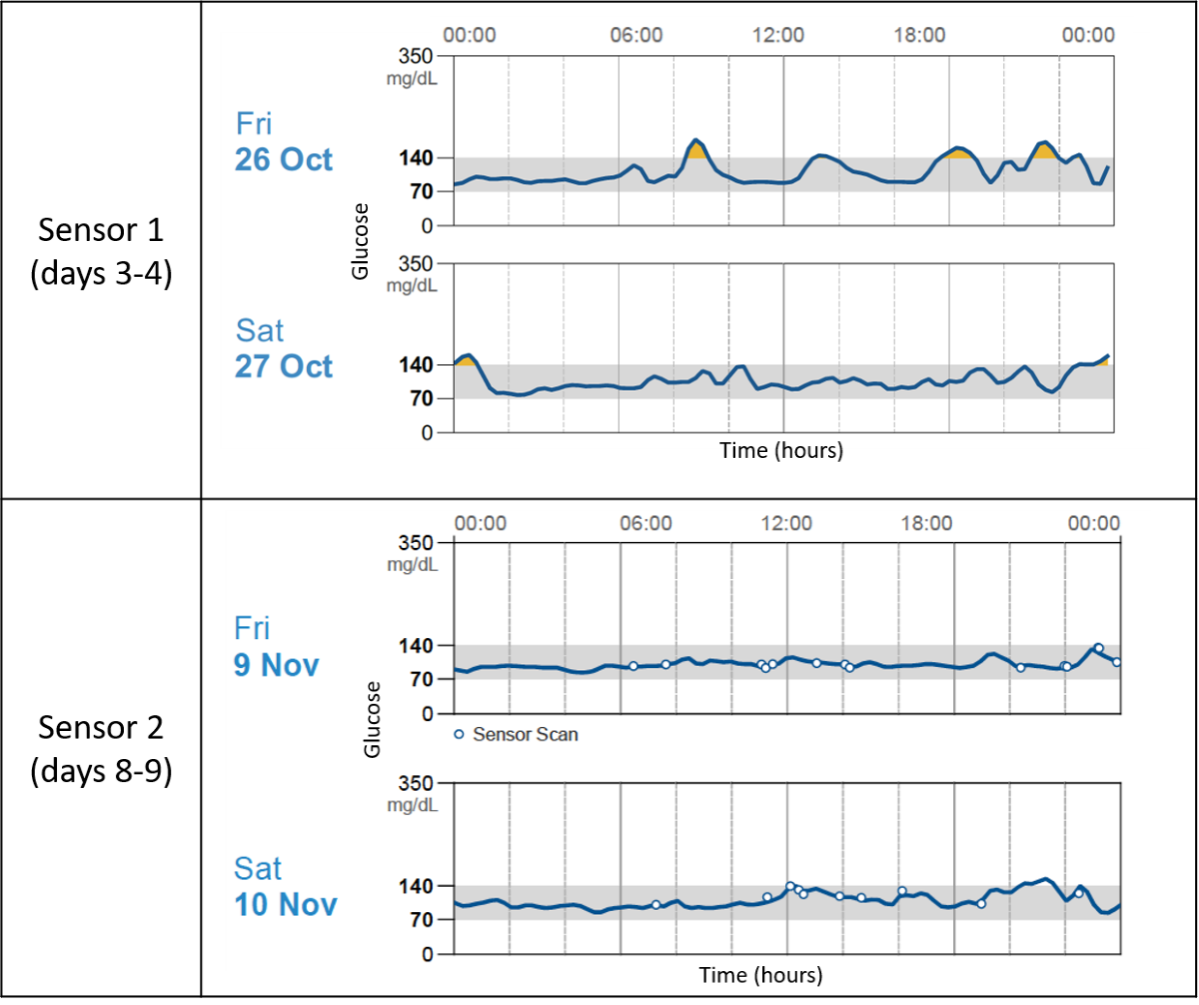
Example of participant glucose trends from both sensor periods captured by Abbott continuous glucose monitor software.

Many participants not only understood the impact of carbohydrates on their blood glucose level but also modified their behavior based on the CGM data. For instance, 1 participant explained their blood glucose trends, a sample of which is also depicted in Figure 3:

I knew when I really binged out. It [the glucose level] went way up, and then crashed, it never went out of that range, but you could still see the crash. I realized, oh, this is what they’re talkin’ about. I would know that that’s not a good way to eat, and I would change my thinking process for my next decision on what I was eating.

Participant 144, Interview 1

For others, seeing the real-time data was reassuring, as it confirmed that they were making better choices:

I didn’t adjust anything, it’s just that I thought this is better for me to see instantaneously. I had that instant gratification of ‘Oh, okay. It’s 93. Oh, okay. It’s 120. Okay, I’m doin’ good.

Participant 153, Interview 1

In the interviews conducted 6 months after the intervention, participants continued to reflect on their experience of wearing the CGM, even though they were not presently wearing one:

The visual was good to see how your body responds to what you eat. And then I guess the lesson in all of that is, to be able to continue to make good choices when you’re not hooked up.

Participant 199, Interview 2

#### Mixed Methods Results

We compared the thematic results of different groups of participants based on significant quantitative results: reduction in HbA_1c_ and weight loss. First, we compared the experiences reported by participants who had a less-than-average reduction in HbA_1c_ levels (<0.71%; n=6) with those reported by participants who had an above-average reduction in HbA_1c_ levels (≥0.71%; n=7). This analysis revealed that regardless of the amount of HbA_1c_ reduction, participants reported that using CGM data to visualize changes in their blood glucose and learning how different foods affected their body was beneficial. All participants reported paying more attention to their blood glucose trends. To illustrate, below are 2 representative quotes about visualizing changes in glucose trends from participants on either end of the HbA_1c_ range.

For example, from the participant with the highest amount of HbA_1c_ reduction during the intervention period:

[The CGM] kinda made me more aware. Like yesterday was a Friday, I probably went out and had a piece a cheesecake and… um, like once in a while I’ll have wine or something. So, I would actually like see it, what that did to my blood sugar, like how it spiked it up. But if I stayed within the parameters, I was fine. It was just on the nights that I go out or something, or the family gets together, that’s when I notice it spikes up and down. 

Participant 105, Interview 1

**Figure 4:**
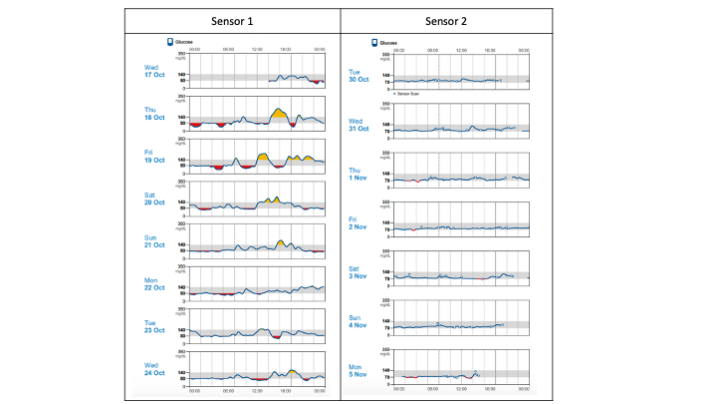
Comparison of daily glucose trends captured by Abbott continuous glucose monitor for one participant from both sensor periods of the intervention.

The spikes that this participant described are evident in the CGM data (Figure 4). During the first week of wearing the sensor (before implementing the low-carbohydrate diet), this participant had blood glucose levels with significant variation and episodes of hyperglycemia. In the second sensor period, while implementing the low-carbohydrate diet, the glucose variability decreased significantly.

A participant with a lower reduction in HbA_1c_ similarly described feeling reassured by being able to visualize the impact of carbohydrates on blood glucose trends:

Even on the day that I had the cookies and stuff. I could see it did go up, and that was like whoa, yeah. There’s a reason for that. Um, and then it came back down. So I could actually see what was happening. But the thing is, I wasn’t eating the potatoes, I wasn’t eating the pasta, and that was pretty much the norm for me. So I didn’t see it go up. But that was a pleasant thing. So it kinda reinforced to me that yes, I am doing the right thing. And yeah I am okay sticking with this, and doing everything kind of according to the book.

Participant 102, Interview 1

Second, we compared the experiences of those who had lost an above-average amount of weight during the intervention (>1.41 lbs; n=7) with those who had lost less than average or gained weight (n=6). Participants who had lost above-average weight often described the diet as easier than the other diets they had tried. In addition, they began to see positive results, including weight loss and feeling better physically. These participants described that they were planning ahead and being more intentional. In contrast, those who gained weight or lost less-than-average weight often had more difficulty with the diet for various reasons, including challenges eating a low-carbohydrate diet during holidays, work events, and family events where the environment is less controlled. Other challenges included giving up old habits and dealing with the emotional aspects of dieting. A comparison of participant experiences according to their average weight change is highlighted in the joint display (a mixed methods strategy for depicting integrated analysis and findings) in Table 4.

**Table 4:** Joint display comparing trial outcomes with patient experiences.

Weight change and corresponding participant experience		Illustrative quote
**Above-average weight loss**		
	Diet was easier to implement, motivated to eat well	* It’s been easy to be honest with ya. You know, I don’t crave bread, I don’t eat bread, I eat a lot more vegetables, very, very little fried food. I mean, it’s been easy . . . And once I walked around with that thing in my arm and I was scannin’ myself every day that just that put a whole new perspective on it.* [Participant 169, Interview 1]
	Saw positive changes	* I’m sleeping better at night. I have more energy I’m in more control of myself, meaning, you know, it’d be like when I was bored, oh, I’d go to the cupboard or something, and just get a little bit of this or a little bit of that. But now I just feel like I eat 3 to 4 times a day and that’s all I need.* [Participant 102, Interview 1]
	Planning or intentionality	* So right now I’m kind of finding my… baseline on what my triggers are. I haven’t really been counting carbs, but I did reach out to my doctor, and I started using continuous glucose monitors, to see if it would help me (Um-hm) So, modified, I guess, yes. I… I’m not necessarily counting carbs, but I’m seeing which foods spike my sugar right now. I’ve only had it in for maybe 4 days. *[Participant 193, Interview 2]
**Below-average weight loss**		
	Difficulty implementing diet	* It’s just that to me a low carb diet is a hard diet . . . and they’re hard to cook. I mean you know, people like me, I’m used to eating pasta, potatoes, you know stuff like that. And those things are easy, quick, you know.* [Participant 141, Interview 1] *I don’t do really well on low carb. When I go no carb or low carb, I don’t do well. My brain does not do well without [carbs]. I just don’t do well. I get emotional… I was crying, I was having mood swings. *[Participant 144, Interview 2]
	Culture of food	* [There were] different parties and different things that I had at work that involved eating. Meetings where I had to take people out for lunch when we get a new hire, and a lot of times you can’t really find things that fit specifically into what you’d like [to eat].* [Participant 179, Interview 2]
	Hard to change habits	* You have to break your whole habits. I think from what I’ve seen it’s probably a good diet, but it’s definitely not easy . . . I mean, we’re… well I’m 71, my wife’s 71, my daughter’s 45, and we’ve… lived like this for a long time. So to just up and change everything is hard. *[Participant 141, Interview 1]

## Discussion

### Principal Findings

We investigated the feasibility of using CGMs combined with low-carbohydrate diet coaching for a dietary intervention in patients with prediabetes. Overall, we found that using CGMs and low-carbohydrate diet coaching is a feasible and acceptable modality for supporting behavior change. All 15 participants wore the CGM sensors and attempted a low-carbohydrate diet during the intervention. Mixed methods results indicated that participants were overwhelmingly satisfied with the intervention and no major adverse effects were noted. Of the secondary outcomes, the reduction in HbA_1c_ and weight loss were significant. Interviews revealed that participants used the data from their CGM to understand the impact of foods with varying quantities of carbohydrates on their body.

Our findings suggest that the use of CGM with low-carbohydrate diet coaching may lead to a reduction in HbA_1c_ and weight loss in patients with prediabetes. Overall, participants described changing their eating behavior as a result of seeing their CGM data, either during low-carbohydrate diet coaching sessions or while receiving real-time feedback from the CGM. These findings are consistent with previous studies conducted on patients with T2DM, where participants who used CGMs for real-time blood glucose readings had greater reduction in HbA_1c_ and glycemic variability than the control group [32]. In this study, participants reported making immediate changes to their next meal because they could see trends and predict how certain foods would affect their blood glucose levels. Despite this, there was no difference in estimated HbA_1c_ between Sensor 2 and Sensor 1. This may have been due to the Hawthorne effect, where wearing the blinded sensor caused participants to consume a diet lower in carbohydrates during the first week of the intervention than they normally would because their blood glucose was being monitored. Further research is needed with a longer intervention period to evaluate the impact of this intervention on individuals with prediabetes.

Others have similarly found that patients with T2DM view CGM technology as an efficient tool to visualize blood glucose readings, monitor trends, and prompt dietary change [33]. Our study is unique as it combines CGM use with low-carbohydrate diet coaching. As carbohydrates drive fluctuation in blood glucose and therefore the trends visible in CGM data, coaching with CGM data provides patients with direct personalized feedback about their carbohydrate consumption. A larger trial studying the independent effects of low-carbohydrate coaching compared with those of CGMs would be valuable to evaluate the synergy of the 2 components of our intervention.

Our findings suggest that an approach combining low-carbohydrate diets and real-time CGM feedback is an acceptable and feasible approach to dietary change among patients with prediabetes. Although exploratory, the mixed methods analysis revealed that participants with the most weight loss had an easier time implementing the diet with intentionality, planning, and motivation. Participants who had the least amount of weight loss or gained weight described more barriers, particularly in breaking old habits or the *culture of food* around them. This is consistent with results of previous qualitative research on barriers (eg, social expectations, financial constraints) and facilitators (eg, motivation to prevent diabetes) to dietary change in patients with prediabetes [34]. Low-carbohydrate diet coaching with CGM feedback may be particularly helpful in supporting participants with prediabetes to maintain motivation or overcome barriers. For example, some participants in our study felt motivated by seeing the reduction in variability (ie, more *time in range*) in their CGM data. In the future, additional coaching that supports participants to set small goals to reduce glucose variability may help to increase motivation. Future investigations of low-carbohydrate diet coaching may also explore the ability of coaching to overcome barriers, including breaking old habits and navigating social events when implementing a low-carbohydrate diet.

Previous research has demonstrated that people with prediabetes underestimate their risk of developing diabetes [24]. In our study, after the intervention, participants felt more reassured that they did not have diabetes, which is likely due to the intervention educating them about their prediabetes status. In addition, they felt that they had a lower risk of developing diabetes in the next 3 years, more personal control, and increased optimistic bias after completing the intervention. When considered alongside the qualitative results, these findings suggest that participants may feel confident that they can maintain positive changes during the intervention, such as weight loss, reduction in estimated HbA_1c_, and time spent *in range* in their CGM data. As the knowledge of prediabetes [35] and perceived risk of developing T2DM [36,37] have been associated with self-care in individuals with prediabetes, further research should investigate the role that CGM data and low-carbohydrate diet coaching may play in influencing these variables.

### Limitations

The primary aim of this pilot study was to assess feasibility. However, with the small sample size and short duration of the study, results must be interpreted cautiously. Given small changes in estimated HbA_1c_ and weight, the results may be due to measurement error. In addition, the duration of the intervention was a total of 22 days, and short-term effects of weight loss may be expected with motivated individuals meeting with the study coordinator every 11 days. HbA_1c_ was not reassessed at enrollment due to the scope of the pilot and feasibility study and was used as the baseline HbA_1c_ for participants. Although our results indicated a significant decrease in HbA_1c_ during the intervention, we used an estimated HbA_1c_ level from CGM data rather than a laboratory test. The estimated HbA_1c_ has fallen out of favor due to inaccuracy [38] and may overestimate changes during the short intervention period. However, estimated HbA_1c_ and the corresponding CGM tracings can be helpful for educational purposes, including understanding how foods differentially impact blood glucose or how physical symptoms (eg, fatigue, low mood) may be related to variations in blood glucose levels [38]. In addition, our pilot and feasibility study did not formally assess low-carbohydrate diet adherence with grams of carbohydrates or grading food logs. Finally, our study sample was comprised primarily of White, female participants. Further research is needed to generalize these preliminary pilot and feasibility findings to other participants with prediabetes.

### Conclusions

The use of CGM feedback with low-carbohydrate diet coaching is feasible for adults with prediabetes, and participants were satisfied with their experience. This novel method deserves further exploration as most studies have focused on CGM use among patients with T2DM rather than use of this device alongside dietary coaching to drive behavior changes to prevent diabetes. Despite the high efficiency of CGM use, there are still barriers that may limit its clinical applications, including provider knowledge of CGMs and out-of-pocket costs for patients. Further research should be conducted to investigate how CGM technology and low-carbohydrate coaching can be used synergistically to prevent diabetes. Future studies are needed to explore the specific mechanisms that support behavior change, including the impact of CGM technology and low-carbohydrate diet coaching on participant knowledge, engagement, and motivation. In addition, more knowledge about sustainability and long-term impact is needed. As the cost of CGM decreases and the technology becomes more ubiquitous, this may become an important strategy for diabetes prevention.

## Abbreviations

CGM: continuous glucose monitor
DPP: Diabetes Prevention Program
HbA_1c_: hemoglobin A_1c_
RPS-DD: Risk Perception Survey for Developing Diabetes
T2DM: type 2 diabetes mellitus
